# Variations in Practice to Therapeutic Monitoring of Tacrolimus following Primary Adult Liver Transplantation

**Published:** 2016-02-01

**Authors:** B. V. M. Dasari, J. Hodson, A. Nassir, J. Widmer, J. Isaac, H. Mergentel, P. Muiesan, D. F. Mirza, M. T. P. R. Perera

**Affiliations:** 1 The Liver Unit, Queen Elizabeth Hospital Birmingham, United Kingdom; 2 Department of Statistics, Wolfson Computer Laboratory, Birmingham, United Kingdom; 3 Consultant Transplant and HPB Surgeon, Birmingham, United Kingdom

**Keywords:** Tacrolimus, Drug monitoring, Immunosuppressive agents [Pharmacological action], Transplantation, Outcome assessment (Health care), Patient outcome assessment

## Abstract

**Background:**

There is limited clinical evidence evaluating the correlation between immunosuppressant monitoring practice and transplant outcomes.

**Objective:**

To assess current practice of tacrolimus trough monitoring in early post-operative period following liver transplantation (LT), and its impact on outcomes.

**Methods:**

The duration to trough levels (DTT) were calculated in patients undergoing primary LT. The impact of variability in DTT on graft rejection episodes, serum tacrolimus level and renal function was assessed. These results were converted into a drug level estimation tool, which was validated in a prospective cohort of patients.

**Results:**

2946 events in 274 patients were evaluated. The median DTT was 7:19 hrs (range: 27 min to 19:38 hrs). In 72% (2140 events) of the occasions, DTT was <8 hrs. There was a significant (p=0.022) correlation between DTT and tacrolimus level. Despite clinical decisions were taken to modify the dose of tacrolimus based on trough level, neither did DTT affect the average creatinine levels (p=0.923), nor the variability in DTT did affect acute rejection (p=0.914, and 0.712, respectively). A dose estimation tool was developed and applied to validation cohort (n=612), and returned a moderate R^2^ value of 0.50.

**Conclusion:**

There is a significant variation in the “real world” monitoring of tacrolimus with DTT in majority of measurements falling below recommendations; reassuringly, this did not lead to adverse transplant sequelae.

## INTRODUCTION

Tacrolimus is a macrolide calcineurin inhibitor used in most immunosuppressant regimes following solid organ transplantation. It was introduced to transplant practice in early 1990s and since then, the efficacy of tacrolimus as a potent calcineurin inhibitor, compared with other drugs of same class has made a significant difference in the long-term graft survival outcomes [1]. Patient and graft survival rates of 75% to 100% and 70% to 95%, at six months and 30 months, respectively, after transplantation were reported with tacrolimus-based immunosuppressive therapy [2]. It acts by binding to intracellular proteins of T cells, inhibiting calcineurin phosphatase, that prevents activation of the nuclear factor of activated T cells, a transcription factor needed for the production of cytokines such as interleukin 2 (IL-2) and γ-interferon [3].

Tacrolimus has a narrow therapeutic index with significant side effect profile that includes nephrotoxicity (50%), hypertension (50%), post-transplant diabetes (15%), neuropathy (30%) and hyperkalemia [4]. In order to prevent complications and maintain the benefits, a therapeutic range of blood levels is aimed. However, the complex pharmacokinetic, pharmacogenetic and pharmacodynamic profile make it challenging to obtain these targeted levels. Laskow and colleagues reported rejection and toxicity rates of 34% and 0%, respectively, in renal transplant patients with tacrolimus levels of 0–5 µg/L and 17% and 34% with levels of 5–15 µg/L [5]. In post-transplant patients, tacrolimus is prescribed as twice daily dosing; however, newer long-acting single dose preparations are available. Generally, trough levels are monitored regularly to determine the dose, and there are various described methods available for therapeutic monitoring. Therapeutic drug monitoring can be performed by using the trough levels, C_max_, C_min_, C_0_, C_2_, AUC levels based on multiple drug concentration levels, or by Bayesian forecasting [6]. The relationship between a range of AUC values and clinical outcomes is not clear [7]. 

The protocol at our unit is to monitor within two hours before the next dose in post-liver transplant (LT) patients. However, in practice, the duration to trough levels (DTT) can be influenced by logistical factors, such as the availability of nursing staff to dispense the medication, and of a junior doctor or phlebotomist in the morning to take the blood samples. This issue is particularly pronounced in a busy transplant unit. These logistical aspects may impact on the trough levels, if the duration between the last dose and the blood samples were either too long or too short. Therefore, the trough levels monitored according to the current practice may not always reflect “optimal” trough levels. We hypothesized that the variations in the DTT lead to “sub-optimal” troughs that might result in an under-/over-dosing, thereby affecting the clinical outcomes. Moreover, there is sparse data in the literature on this aspect in the clinical transplant setting; hence, we set up this study to assess the current practice of drug monitoring of tacrolimus in the early post-operative period following LT.

## PATIENTS AND METHODS

All patients who underwent the first LT operations between January 2011 and January 2013 were included in the study. Those who had redo-LT and, thereby, had previous exposure to tacrolimus, were deemed incomparable to those undergoing the procedure for the first time and were excluded from the study. Records of all patients admitted during this period were available on the hospital electronic database, the prescribing information and communication system (PICS) [8]. This electronic database is diligently managed, with all the details of the patients from the time of hospital admission to discharge. Patient observations, in-patient prescriptions, time of drug dispensation and time and type of intervention are documented and are the only source of data for patient monitoring and management within the hospital. Patient consent was obtained pre-operatively for all the investigations performed in this study. 

Dosing and Blood Sampling

In our unit, oral tacrolimus is prescribed twice daily at 10:00 hrs and 22:00 hrs. Blood samples for trough levels are taken between 08:00 hrs and 10:00 hrs, six days a week. Trough levels, liver and renal function tests are available for the evening ward round when the drug dosage is adjusted, according to need on a per patient basis, in order to achieve the appropriate blood tacrolimus levels.

Definitions of Outcome

DTTs were calculated based on the time drug was dispensed to patient, to the time a blood sample was taken following morning. For the purpose of this study, renal impairment was defined as a rise in baseline serum creatinine by >26 µmol/L on at least two separate or consecutive measurements [9]. While the rise in creatinine suggests acute kidney injury, the significant variations encountered due to fluid imbalance or other nephrotoxic drugs in this group of patients were accounted by looking for the rise in creatinine on at least two occasions. Outcomes assessed were the effect of DTTs on the subsequent tacrolimus levels, on subsequent creatinine levels, and immunological outcomes in terms of biopsy-proven graft rejection. 

Data Analysis

All variables relating to tacrolimus levels/doses and creatinine were found to have skewed distributions, and so were log_2_-transformed prior to the analysis, in order to meet the assumptions of parametric tests. In order to account for the fact that repeated measures on a patient were likely to be correlated, the data were analyzed using generalized estimating equations (GEEs), with an autoregressive correlation structure. In these models, measurements from the previous day were included as factors, as these were likely to have a significant influence on measurements made on the following day. Due to the log-transformations, the resulting coefficients related to the log_2_ of the outcome. Therefore, the coefficients were anti-logged and converted into a percentage change in the outcome for ease of interpretation. Where the factors were also logged, the coefficients represent the percentage change in the outcome for a two-fold increase in the level of the factor. 

To consider the impact of DTT on rejection, the mean DTT for each patient was first calculated. The resulting values were compared between those patients with a rejection episode and those without, using an independent samples *Student’s t *test. The process was repeated using the standard deviation in DTT for each patient, to test whether patients with highly variable DTT were more likely to have rejection. A parsimonious model was then produced for the prediction of tacrolimus levels, with the intention of creating a dose estimation tool. This was then applied to a validation cohort of patients, in order to assess whether it was sufficiently accurate to be of use practice. All analyses were performed using IBM SPSS^®^ 19, with p<0.05 deemed to be indicative of statistical significance.

## RESULTS

A total of 274 patients underwent first LT operations during the study period. The basic demographics of this patient cohort is summarized in Table 1. From these patients, 2946 events of tacrolimus monitoring were available for inclusion in the analysis. Complete consecutive data sets for tacrolimus and creatinine levels for model predictions reported below were available in 1521 and 1449 events, respectively. Biopsy-proven rejection was diagnosed in 67/274 (24.4%) whilst renal impairment was noted in 130/274 (47%) patients. 

**Table 1:** Characteristics of the patients included in the study. Values are n (%) or median (range).

Patient Characteristics	Statistics
Sex	
Male	168 (61.3%)
Female	106 (39.4%)
Ethnicity	
White	245 (89.4%)
Asian British	21 (7.7%)
Afro-Caribbean	7 (2.6%)
Mongoloid	1 (0.4%)
Age in years (median/range)	57.3 (17.3–73.4)
BMI (median/range)	28.1 (17.1–43.8)
Pre-operative renal impairment	27 (9.9%)
Pre-operative diabetesmellitus	40 (14.6%)
Pre-operative bilirubin (median/range)	20 (5–3353)
Creatinine µmol/L (median/range)	74 (20–687)
INR (median/range)	1.5 (0.9–38)
Primary diagnosis	
Alcohol cirrhosis	67 (24.4%)
Hepatitis B and hepatitis C cirrhosis	54 (19.7%)
Primary biliary cirrhosis	39 (14.2%)
Primary sclerosing cholangitis	30 (10.9%)
Acute liver failure	17 (6.2%)
Cryptogenic cirrhosis	12 (4.3%)
Others	55 (20.0%)

Duration to Trough Monitoring

**Figure 1:**
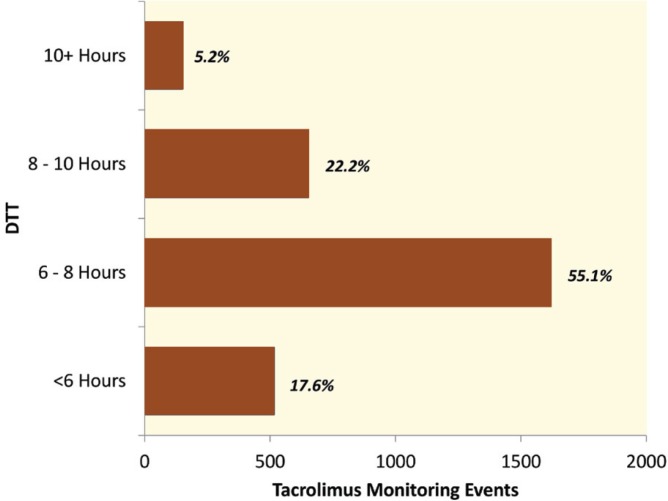
Percentage of tacrolimus monitoring events *vs*. duration to trough (DTT

In the entire cohort, the median DTT was 7:19 hrs (range: 27 min to 19:38 hrs). In nearly 75% (n=2140) of occasions out of 2946 events, the tacrolimus levels were monitored under eight hours of last dose; with DTTs <6 hours in 518 (17.6%) and between 6 and 8 hours in 1622 (55.1%) events. Furthermore, the DTT came closest to manufacturer recommendations and monitored between 8 and 10 hours in 654 (22.2%) and >10 hours in 152 (5.2%) events (Fig 1).

Effect of DTT on Tacrolimus Levels 

**Figure 2:**
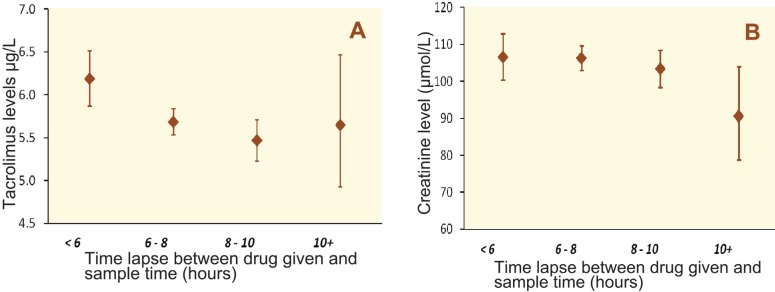
Variation in the mean tacrolimus levels (A) and mean creatinine levels (B) *vs*. DTT, from the model presented in Table 2

Tacrolimus levels were found to decrease progressively with increasing DTT, from 6.18 µg/L (95% CI: 5.87 to 6.52) at <6 hours, to 5.68 (95% CI: 5.53 to 5.84) at 6–8 hours, and 5.47 µg/L (95% CI: 5.23 to 5.72) at 8–10 hours (Fig 2A). A small increase to 5.65 µg/L was observed for DTT of 10 or more hours; however, the confidence interval for this geometric mean was very wide due to the small number of monitoring events with a delay of this size (95% CI: 4.93 to 6.47). The GEE analysis (Table 2) found that both the previous day’s tacrolimus level and dose were significant predictors of the current day’s tacrolimus levels (both p<0.001). After accounting for this, the effect of DTT remained significant (p=0.002) with tacrolimus levels being 6.8% lower (95% CI: 2.0% to 11.4%; p=0.006) for a DTT of 6–8 hours, and 8.1% lower (95% CI: 1.9% to 14.0%; p=0.012) for a DTT of 8–10 hours, relative to DTT <6 hours. 

**Table 2:** Results from the generalized estimating equation model predicting tacrolimus levels. Coefficients represent the percentage increase in tacrolimus relative to the reference category, unless started otherwise

Factor	Coefficient (95% CI)	p value
DTT (hours)		0.022
<6	—	—
6–7.99	6.8% (11.4% to 2.0%)	0.006
8–9.99	8.1% (14.0% to 1.9%)	0.012
≥10	0.4% (11.7% to 12.4%)	0.951
Previous day tacrolimus level*	49.7% (45.5% to 54.0%)*	<0.001
Previous day tacrolimus dose*	8.7% (5.6% to 11.8%)*	<0.001

* Coefficient represents the percentage increase in tacrolimus for a two-fold increase in the factor.

Effect of DTTs on Creatinine Levels

The mean creatinine levels across the categories of DTT are illustrated in Figure 2B. The average creatinine levels were found to be similar across the DTT groups, with geometric means of 106.4 µmol/L at <6 hours (95% CI: 100.4 to 112.8), 106.1 µmol/L at 6–8 hours (95% CI: 102.8 to 109.5) and 103.2 µmol/L at 8–10 hours (95% CI: 98.3 to 108.4) (Fig 2B). A small decline was observed for DTT of 10 or more hours (90.4 µmol/L; 95% CI: 78.7 to 104.0), although the confidence interval was very wide due to the small number of patients. GEE analysis (Table 3) found that after accounting for the significant relationships between the previous day’s creatinine level and tacrolimus dose (both p<0.001), there was no evidence of a significant relationship between creatinine and DTT (p=0.923).

**Table 3:** Results from the generalized estimating equation model predicting tacrolimus levels. Coefficients represent the percentage increase in creatinine relative to the reference category, unless stated otherwise

Factor	Coefficient (95% CI)	p value
DTT (hours)		0.923
<6	—	—
6–7.99	0.6% (3.8% to 2.7%)	0.725
8–9.99	1.1% (5.2% to 3.2%)	0.611
≥10	1.9% (7.6% to 12.4%)	0.708
Previous day tacrolimus level*	1.1% (0.5% to 2.6%)*	0.170
Previous day tacrolimus dose*	2.2% (0.9% to 3.5%)*	<0.001
Previous day Creatinine*	92.5% (89.7% to 95.3%)*	<0.001

* Coefficient represents the percentage increase in creatinine for a two-fold increase in the factor.

Effect of DTTs on Rejection

The mean DTTs were not found to differ significantly between those patients with (7.39 hours, 95% CI: 7.17 to 7.60) or without (7.37 hours, 95% CI: 7.24 to 7.50) rejection (p=0.914). The standard deviation of DTT was also calculated for each patient. There was no evidence that these values differed significantly between those patients with (1.57 hours, 95% CI: 1.34 to 1.80) and without (1.51 hours, 95% CI: 1.37 to 1.66) episodes of rejection (p=0.712).

Prediction of Tacrolimus Levels

A parsimonious version of the GEE model of tacrolimus levels was produced, with the intention to create a dose estimation tool. The resulting formula is:

Tacrolimus Level = 2 ^1.06+0.56^^×^^PTL+0.14^^×^^PTD-d^

where,

PTL=log _2_ (Previous day's tacrolimus level)

PTD=log _2_ (Previous day's tacrolimus dose)

and

d=0.00ifDTT is<6 hours0.09if DTT is<6-8 hour0.13if DTT is<8-10 hours0.01if DTT is≥ 10 hours

**Figure 3:**
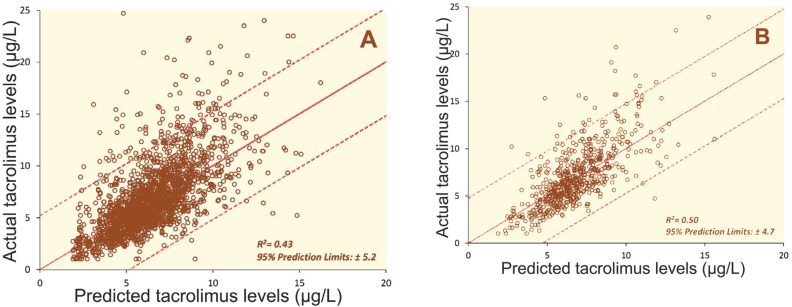
Predicted tacrolimus levels *vs*. actual tacrolimus levels in the modelling cohort (A), and validation cohort (B), based on the dose estimation tool. The solid line indicates the target for equivalence; the broken likes are the 95% prediction limits

This equation was applied to a modelling data (Fig 3A) from this study and was prospectively validated in a cohort of 139 patients, with 612 valid tacrolimus monitoring events (Fig 3B). The validation group included the consecutive adults undergoing primary LT in the unit from February 2013 to December 2013. The predicted measurements were a reasonable fit to the trend of the data. However, there was a relatively wide margin of error, with an R^2^ value of 0.50, and 95% prediction limits of ±4.7.

## DISCUSSION

The clinical use of tacrolimus is complicated by the complex pharmacokinetics, pharmacogenetics and phamacodynamics. Oral bioavailability of tacrolimus is only 20%–25% and elimination half-life has been reported to be approximately 12 hours in LT recipients [10]. Whole blood concentrations reach a maximum within two hours after the dose administration, following which they drop significantly after four hours, followed by a slow plateau [11, 12]. Hepatic metabolism and intestinal efflux via P-glycoprotein is an important route for the drug elimination and contribute significantly to the variation in the pharmacokinetics [11]. Tacrolimus is mostly metabolized by the cytochrome P450 3A4 system (CYP3A4) of hepatocytes. Patients with at least one CYP3A5*1 functional allele (*ie*, CYP3A5 expressers) on average need twofold higher doses than CYP3A5*3/*3 (*ie*, CYP3A5 nonexpressers) to reach the same blood levels [13]. In addition, CYP3A4 system can be inhibited or induced by various drugs such as antiepileptic or antifungal agents. Various factors also influence the pharmacokinetics of tacrolimus, including hepatic dysfunction, hepatitis C infection, renal function, patients’ age, time since transplantation, drug interactions, patients’ sex and ethnicity, and the organ transplanted. Consequently an individually administrated dose does not directly correlate with subsequent blood concentrations. 

However, the actual trough levels of tacrolimus are directly associated with its clinical effects of immunosuppression as well as its side effects. Trough levels <5 ng/mL are insufficient to obtain adequate immunosuppression and >20 ng/mL are assumed toxic. Conventionally, target range of trough levels was 10–15 ng/mL during the first 4–6 weeks with a progressive reduction thereafter, achieving 5–10 ng/mL in the long-term, with minimal variations between studies. A recent systematic review suggests a target tacrolimus trough between 6 and 10 ng/mL during the first 4–6 weeks and a progressive reduction of dosage to achieve a steady state level between 4 and 8 ng/mL in the long-term in order to reduce renal impairment and not to increase moderate to severe acute cellular rejection simultaneously [14]. A European consensus statement on optimization of tacrolimus therapy in solid organ transplantation suggested that AUC concentrations between 150 and 200 ng/h/mL would be appropriate in the management of post-solid organ transplant patients [15].

Therapeutic drug monitoring can be performed by using the trough levels, C_max_, C_min_, C_0_, C_2_, AUC levels based on multiple drug concentration levels, or by Bayesian forecasting. It has been estimated that the trough levels are a reasonable predictor of the AUC with a probability of 0.9 [11, 16], although some reported poor correlation [15]. Limited sampling strategies instead of a single sample are also investigated and reported to have good predictability of AUC concentration [7]. 

About 95% of the drug is bound to the red cells (RBCs) and 5% is in the plasma [17]. Although the free plasma levels reflect more accurate trough levels, in practice, whole blood concentrations are measured using tandem mass spectroscopy. However, the effect of pre-dose tacrolimus levels or that of the AUC on clinical outcomes is still debatable. A significant linear relationship between tacrolimus concentration and adverse events has been reported but not with the rejection rates after LT [15]. Bouamara and colleagues found no impact of pre-dose tacrolimus levels on renal graft rejection rates [18]. Similarly, Capron and colleagues showed that there is no correlation between tacrolimus whole blood concentrations and rejection after LT [19].

Single trough levels are most commonly practiced in the UK transplant units. While the trough levels are recommended at two hours before the next sample, in our study, only a small proportion of the trough levels were compatible with this recommendation. Considering the drug metabolism, trough levels are expected to be significantly higher in those patients with trough levels taken prior to the two hours lag and *vice versa*. This has been reflected in our group of patients, with tacrolimus levels being highest in those with samples taken within six hours following drug administration and a reducing progressively in trough samples taken at 6–8 hours and 9–10 hours. Based on what has been understood with the dynamics of the drug, these trough levels should result in an increased nephrotoxicity in patients with samples taken at 6–8 hours or those taken at <6 hours. However, we could find no evidence that this was the case, as creatinine levels were not significantly affected by the timing of trough levels. This could possibly be explained by the close monitoring of renal functions and adjustment of the tacrolimus dose/levels. 

Acute cellular rejection is diagnosed by histological confirmation after biochemical suspicion of rejection and in patients receiving only tacrolimus and steroids, its incidence was reported to be 25%. Using lower tacrolimus concentrations could result in increased incidence of rejection, especially early after transplantation. In our study, 26% of the patients had histologically confirmed rejection comparable to the rejection rates in the literature (over 40%) [20, 21]. However, neither the average of, nor variability in DTT were found to have any significant effects on rejection rates.

An offshoot of this study was the dose estimation tool to predict tacrolimus levels based on the DTT, and the dose and level on the previous day. Advantages of such a tool would be to predict the appropriate dose required to achieve the therapeutic concentrations that could be more economical way of monitoring by reducing the need for daily monitoring, avoid large fluctuations in the tacrolimus levels, and target dosing to obtain appropriate concentration levels. Although our dose estimation model showed reasonable predictive accuracy, and R^2^ value of 0.50 (Fig 3), there was still a considerable amount of error in some estimates, with the 95% prediction intervals of ±4.7. Due to the toxicity and narrow therapeutic range of tacrolimus, a model that recommends dosing could over- or under-estimate the resulting tacrolimus level by such a margin was considered not safe for use in practice. This variation may be the result of differences in bioavailability and phamacogenetics among patients. The accuracy of the model is affected by additional factors that influence the blood levels of tacrolimus. Although our initial validation demonstrated its limited clinical use, this model paves way for future development of more reliable individualized dose estimation tools. 

In summary, this is the one of the largest studies evaluating the clinical pharmacokinetics of tacrolimus usage in LT and its clinical implications. The study is limited by its retrospective methodology. The study highlights the variations in the practice of tacrolimus monitoring in the current practice and discussion on the way further monitoring protocols should evolve in future. 

## Notes

**FUNDING SOURCE:** None.
